# Microvessel area as a predictor of sorafenib response in metastatic renal cell carcinoma

**DOI:** 10.1186/1475-2867-14-4

**Published:** 2014-01-14

**Authors:** Saadia A Aziz, Joshua A Sznol, Laurence Albiges, Christopher Zito, Lucia B Jilaveanu, Robert L Camp, Bernard Escudier, Harriet M Kluger

**Affiliations:** 1 Departments of Medicine, Section of Medical Oncology, Yale University School of Medicine, 333 Cedar St., WWW213, New Haven, CT 06520, USA; 2 Departments of Medicine and Pathology, Yale University School of Medicine, New Haven, CT, USA; 3 Department of Medical Oncology, Institut Gustave Roussy, Villejuif, France

**Keywords:** Renal cell carcinoma, Microvessel area, Angiogenesis, Sorafenib

## Abstract

**Background:**

Sorafenib was the first Food and Drug Administration approved anti-angiogenic therapy for renal cell carcinoma (RCC). Currently, there are no validated predictive biomarkers for sorafenib. Our purpose was to determine if sorafenib target expression is predictive of sorafenib sensitivity.

**Methods:**

We used an automated, quantitative immunofluorescence-based method to determine expression levels of sorafenib targets VEGF, VEGF-R1, VEGF-R2, VEGF-R3, c-RAF, B-RAF, c-Kit, and PDGFR-β in a cohort of 96 patients treated with sorafenib. To measure vasculature in the tumor samples, we measured microvessel area (MVA) by CD-34 staining.

**Results:**

Of the markers studied, only high MVA was predictive of response (*p* = 0.005). High MVA was associated with smaller primary tumors (*p* = 0.005). None of the biomarkers studied was predictive of overall or progression-free survival. Using the Bonferroni adjustment correcting for 9 variables with an alpha of 0.05, MVA remained significantly associated with sorafenib response.

**Conclusions:**

Our results suggest that high MVA in tumor specimens might be associated with a greater likelihood of response to therapy. Further studies are needed to confirm these results in additional patients and in patients receiving other VEGF-R2 inhibitors, as MVA might be useful to improve patient selection for VEGF-R2 inhibitors.

## Introduction

Despite emergence of new drugs for patients with unresectable or metastatic RCC (mRCC), most therapies are not curative. Response rates are 15-44%, and the five-year survival for mRCC is only 10% [1]. Immunotherapy once represented the standard treatment; responses to interferon-alpha are approximately 12% and typically not durable, whereas response rates to high-dose interleukin-2 are approximately 14%, and often durable [2,3]. Although newer therapies such as Nivolumab are promising, there remains great need for additional therapies, along with predictive biomarkers to improve the therapeutic window [4].

Mutations or silencing of the von Hippel-Lindau tumor-suppressor gene are often found in clear cell RCC, the most prevalent mRCC sub-type [5]. VHL silencing leads to dysregulated hypoxia-induced factors and activation of downstream pathways important for tumor progression [6]. The upregulation of vascular endothelial growth factor (VEGF), platelet derived growth factor (PDGF), and other pro-angiogenic proteins have led to development of therapies targeting angiogenesis and VEGF pathway members in RCC [7].

There is a variety of Food and Drug Administration (FDA) approved targeted therapies for mRCC. These include tyrosine kinase inhibitors (TKIs), sunitinib, sorafenib, pazopanib, and axitinib, which primarily target VEGF receptors. Other drugs include the anti-VEGF antibody bevacizumab given with interferon and mTOR inhibitors, temsirolimus and everolimus [8].

Sorafenib, initially identified as a Raf kinase inhibitor, was the first FDA-approved anti-angiogenic multikinase inhibitor for mRCC. Sorafenib inhibits C-RAF, B-RAF, VEGFR-2, VEGF-R3, PDGFR-β, c-KIT and FLT-3 [9]. The IC_50_ for enzyme inhibition varies, and is low for VEGF-R2. A randomized discontinuation placebo-controlled phase II trial demonstrated prolonged progression-free-survival (PFS) in patients receiving sorafenib [10]. In a randomized phase III trial, the Treatment Approaches in Renal Cancer Global Evaluation Trial (TARGET), sorafenib prolonged median PFS from 2.8 to 5.5 months. Although the initial intent-to-treat analysis did not show a significant overall survival (OS) benefit, a secondary analysis, censoring placebo-treated patients who crossed over to sorafenib, demonstrated a survival advantage for those receiving sorafenib [11,12]. Several biomarkers have been studied as potential predictors of sorafenib response, to improve patient selection. Kusuda et al. assessed the association between expression of 19 molecular markers by immunohistochemistry and response to sorafenib in 45 mRCC patients. Bcl-xL, PDGFR-α, bone metastasis, and c-reactive protein levels were associated with PFS by univariate analysis. On multivariable analysis, PDGFR-α maintained significance [13]. Jonasch et al. evaluated expression and activation of phosphoinositide-3-kinase pathway members in tumors of 22 sorafenib-treated patients and 18 treated with sorafenib/interferon. High pAKT was associated with worse PFS [14]. Using tumor and plasma samples of patients enrolled on the TARGET trial, Peña et al. showed that soluble plasma VEGFR-2 and CAIX, TIMP-1, Ras p21, and VHL mutations in tumors were not predictive of sorafenib response [15]. In 83 mRCC patients treated with sorafenib, a low erythrocyte sedimentation rate was predictive of improved PFS [16]. Zurita et al. demonstrated that low IL-2, IL-5, and monocyte chemotactic protein 1, and high EGF, IL-12 p40, and M-CSF were correlated with shorter PFS [17].

The association between tumor vascularity and response to VEGF and VEGF-receptor targeting drugs has been studied in small series. In pilot studies, vascular permeability decreased after sorafenib treatment, correlating with time to progression (*P* = 0.01). Elevated baseline tumor vascular permeability, defined by Dynamic Contrasted-Enhanced-Magnetic Resonance Imaging (DCE-MRI), correlated well with improved PFS (*P* = 0.003), but not with radiographic decrease in tumor size [18].

Pretreatment prognostic clinical variables which form the MSKCC score have been well established in mRCC. Poor Karnofsky performance status, high serum lactate dehydrogenase (LDH), low hemoglobin, and high serum calcium are associated with poor OS [19]. In the TARGET trial, MSKCC score was an independent predictor of OS in both placebo and sorafenib-treated patients [12]. We studied associations between pre-treatment tissue levels of sorafenib targets and microvessel area (MVA) and sorafenib activity in sorafenib-treated patients. Traditional immunohistochemical analyses are limited by subjectivity and qualitative assessment. We employed a method of automated, quantitative analysis (AQUA) to determine levels of sorafenib targets (B-Raf, C-RAF, cKIT, PDGF-Rβ, VEGF-R1, VEGF-R2, VEGF-R3, VEGF) and MVA [20-22]. We found no correlation between expression of these markers, PFS and OS, although high MVA was associated with a greater likelihood of response.

## Patients and methods

### Tissue microarray (TMA) construction

With approval of institutional review boards at Institut Gustave Roussy and Yale University, we identified 116 sorafenib-treated mRCC patients, of which 96 had ample viable tissue. The majority was enrolled in the TARGET study by the Institut Gustave Roussy, while others were enrolled in the randomized phase 2 front line study, or in the expanded access program, and 7 were treated at Yale after approval of sorafenib [23,24]. Previous treatments included high-dose IL-2, interferon, IL-2 and interferon, interferon and bevacizumab, provera, interferon and velban, cisplatin and gemcitabine, and sunitinib. Nine patients received prior VEGF/VEGFR targeting drugs. Four received sunitinib, three received prior bevacizumab and two received both. TMAs were constructed using 0.6 mm cores spaced 0.8 mm apart. Nephrectomy specimens were used for these analyses, based on tissue availability. In previous studies we showed that MVA and expression patterns of sorafenib targets were not different in metastatic and matched primary tumors [25,26]. Tumors from each patient were represented by three cores from different areas, avoiding areas of necrosis. Demographics, clinical characteristics, MSKCC risk factors and response to sorafenib are summarized in Table 1. Follow up time ranged from two to 87 months, median 14.5 months.

**Table 1:** Patient characteristics

	**Responders**	**Non-responders**	**Chi-statistic**	**p-value**
Sex			0.026	0.8721
Male	14	62		
Female	4	16		
AGE			0.027	0.8688
<=55	10	45		
>55	8	33		
Fuhrman Grade			0.074	0.7862
1-2	4	13		
3-4	10	39		
Tumor Size			1.311	0.2522
<=75 mm	9	23		
>75 mm	5	26		
Performance Status			0.087	0.7682
0	9	36		
>1	9	42		
LDH			0.325	0.5686
Normal	17	64		
Elevated	1	7		
Calcium			1.343	0.2465
Normal	18	66		
Elevated	0	5		
Hemoglobin			0.464	0.4956
Normal	12	53		
Low	6	18		

### Immunofluorescence

Slides were stained individually for target markers; B-RAF, C-RAF, cKIT, PDGF-Rβ, VEGF-R1, VEGF-R2, VEGF-R3, VEGF. Immunofluorescent staining was performed as described [20,27]. CD-34 staining was used to determine MVA, as described [21,22]. MVA was determined by percent area of CD-34 staining within the tumor mask area. Details of antibodies and dilutions are provided in Additional file 1: Table S1 [25,26].

To determine specificity of each antibody lot, we performed immunoblotting to verify binding to a single band at the expected molecular weight (not shown). A number of commercially available VEGF-R2 antibodies were tested. As previously described, the A-3 antibody (Santa Cruz Biotechnologies, Inc, Santa Cruz, CA) was superior in our hands to the 5B11 (Cell Signaling Technologies, Danvers, MA) [26].

### Automated image acquisition and analysis

Image analysis algorithms have been previously described and adapted for RCC tissues [20,22]. The percentage of CD-34 area within the tumor area was used to determine MVA [25].

### Statistical analysis

We used JMP 5.0 software for data analysis (SAS Institute, Cary, NC). Scores for replicate tumor cores were averaged. Associations between biomarkers and binarized clinical parameters were performed by ANOVA. Survival analyses were done using the Cox proportional hazards method, and survival curves generated using the Kaplan Meier method.

## Results

We first explored associations between known clinical prognostic parameters and outcome in these sorafenib-treated patients. Patients were assigned to two groups using RECIST 1.0 (response evaluation criteria in solid tumors) to determine the best response: partial and complete response (PR, CR) or stable and progressive disease (SD, PD). Scans were assessed by the institutional radiologist and the treating oncologist. The percentage of patients with PR and CR was higher than typically seen in sorafenib-treated patients [11]. We also studied the association between established prognostic markers and PFS and OS. Lung metastases were associated with a greater likelihood of response to sorafenib (χ^2^ = 3.953, *p* = 0.0468) and prolonged PFS (*p* = 0.0317), while metastases in other locations were not associated with response or survival. Patients with low hemoglobin had shorter PFS, as did patients with poor performance status (log rank *p* < 0.0001 for both). High LDH, advanced age, and male gender were associated with shorter PFS, but this was not statistically significant. Clinical variables associated with worse OS included bone metastases (*p* = 0.0051), low hemoglobin (*p* = 0.0024) and poor performance status (*p* < 0.0001).

**Figure 1:**
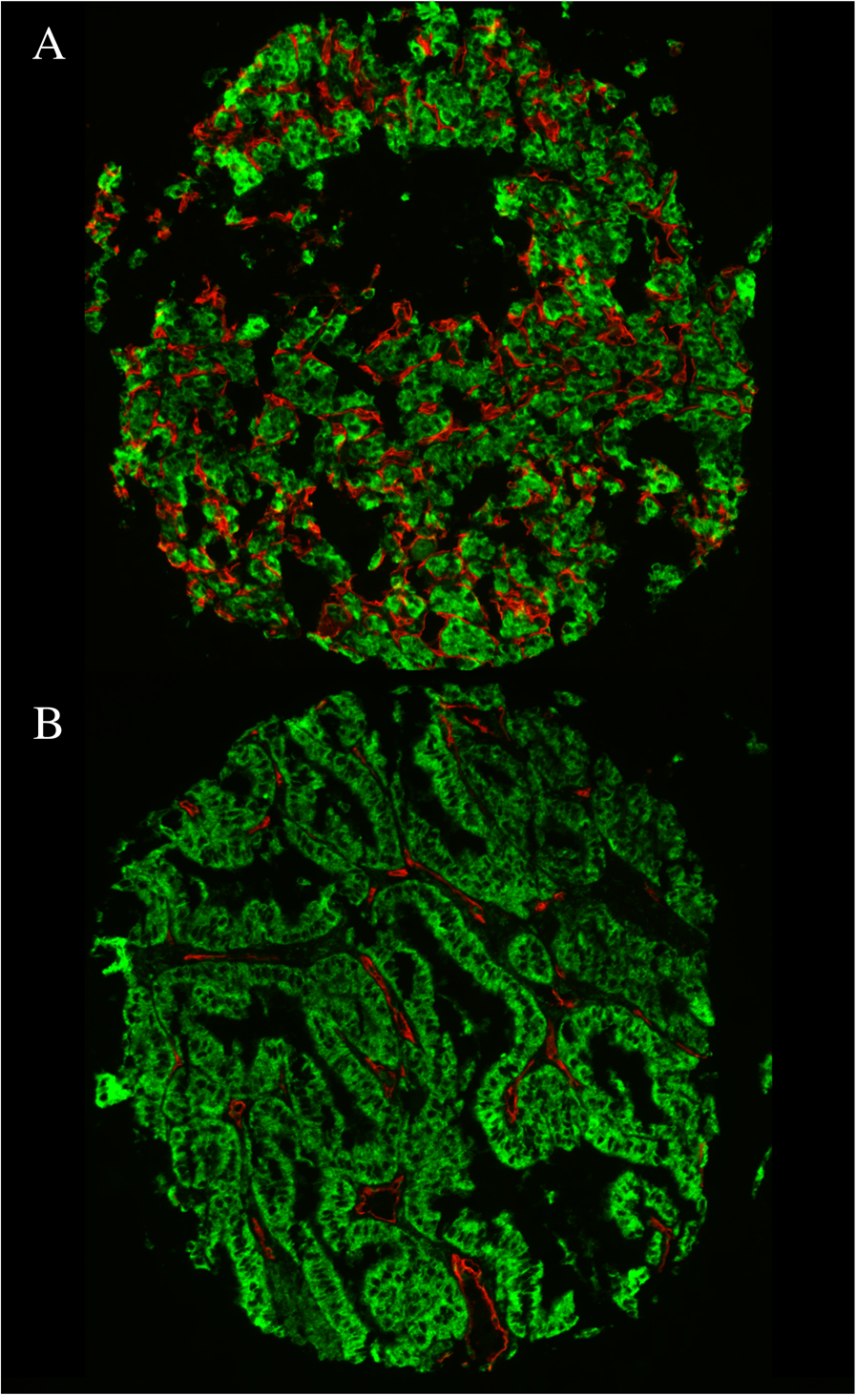
**High (panel A) and low (panel B) microvessel area (MVA) by AQUA.** We used a cocktail of anti-cytokeratin and anti-carbonic anhydrase-9 conjugated to Cy2 to create a tumor mask (green), and anti-CD-34 conjugated to Cy5 (red) to identify microvessels. An example of a patient with high MVA is shown in panel **A**, and an example of low MVA in panel **B**. The corresponding MVA scores were 28.37% and 3.31%.

We next determined the association between biomarker expression and response to sorafenib. Additional file 1: Table S2 contains AQUA score distributions. Figure 1A shows an example of a highly vascular tumor, stained with anti-CD-34, Figure 1B a less vascular tumor.

**Figure 2:**
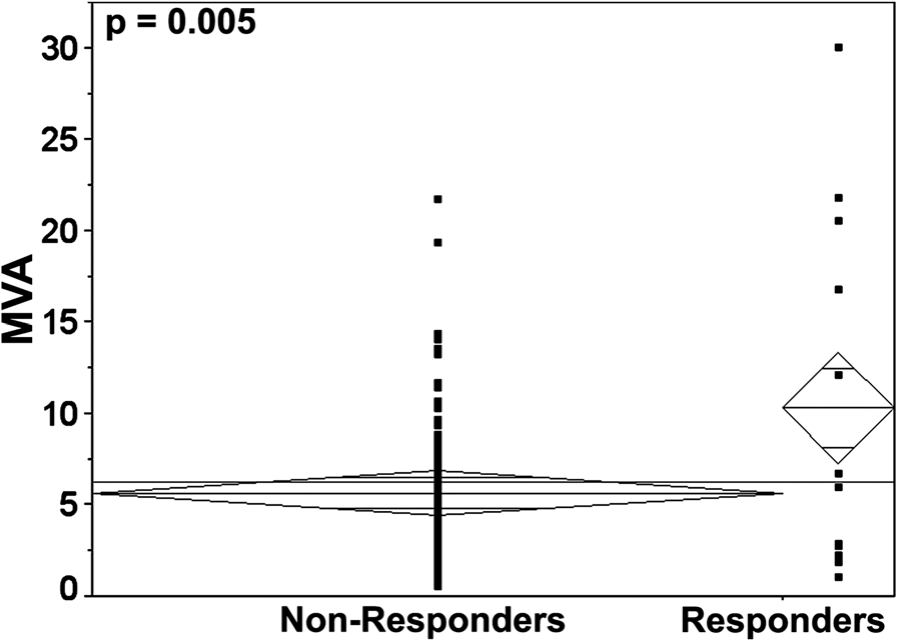
Means plot analysis depicting differences in MVA between sorafenib non-responders (progressive disease and stable disease) and responders (partial and complete responders).

We measured the association between continuous AQUA scores for each marker and sorafenib response using a two-sample *t-*test. High MVA was associated with a greater likelihood of response (*p* = 0.005) (Figure 2). This association was independent of other known predictive markers (performance status, LDH, calcium and hemoglobin). Expression of sorafenib targets in tumor cells was not correlated with response (Table 2). We dichotomized continuous AQUA scores into high and low expressers by the median for each marker. High C-Raf was associated with improved OS but not PFS (data not shown). No other markers were significantly associated with either PFS or OS. Using the Bonferroni adjustment correcting for 9 variables with an average inter-variable correlation of 0.4, an alpha of 0.05 is reduced to 0.013. Consequently MVA remained significantly associated with sorafenib response.

**Table 2:** Associations between markers and response to sorafenib

	**Mean +/- STD**								
**Marker**	**Responders**			**Non-responders**			**Delta**	**t-statistic**	**p-value**
**MVA**	**10.3**	**+/-**	**9.7**	**5.6**	**+/-**	**4.2**	**4.7**	**2.847**	**0.0055**
VEGF	33.4	+/-	11.2	38	+/-	10.9	-4.6	-1.548	0.125
VEGF-R1	32.4	+/-	7.6	33	+/-	8.2	-0.6	-0.288	0.7739
VEGF-R2	31.4	+/-	8.3	33.4	+/-	8.0	-2	-0.929	0.3554
VEGF-R3	48.3	+/-	13.3	50.9	+/-	10.2	-2.6	-0.879	0.3817
c-RAF	35.1	+/-	14.9	34.4	+/-	13.5	0.7	-0.177	0.8601
B-RAF	39.5	+/-	12.8	42	+/-	14.1	-2.5	-0.707	0.4813
c-Kit	44.0	+/-	12.8	43.8	+/-	12.4	0.2	-0.047	0.9629
PDGFR-β	37.5	+/-	14.9	33.6	+/-	9.5	3.9	-1.321	0.1897

We then determined whether marker expression or vascularity was associated with other clinical/pathological characteristics by ANOVA, including age at diagnosis (binarized at 50 years), gender, primary tumor size (dichotomized by median size, 75 mm), and Fuhrman Grade (I/II versus III/IV). High MVA was associated with small primary tumors (*p =* 0.0273). Associations between marker expression and prognostic variables are shown in Additional file 1: Table S3. High tumor VEGF-R2 and PDGF-Rβ were associated with poor performance status (*p =* 0.043 and *p* = 0.023). High VEGF was associated with high LDH (*p =* 0.032). High tumor VEGF-R2, high VEGF-R3 and high PDGF-Rβ were associated with low hemoglobin (*p =* 0.006, *p =* 0.036, *p =* 0.044, respectively). The number of patients with elevated LDH and calcium was small. For all other markers, there was no significant association between expression and clinical variables.

## Discussion

Here we quantified intensity of sorafenib target expression and determined vessel area in nephrectomy specimens of mRCC patients treated with sorafenib. MVA in nephrectomy was predictive of sorafenib response. Expression levels of direct sorafenib targets were not associated with response or PFS. MVA was also associated with small primary tumors. The cohort of specimens available to us was enriched for patients who achieved a response; 19% had either a partial or complete response, whereas of the 451 sorafenib-treated patients in the TARGET trial, 44 (10%) responded [11]. This response rate is similar to that recently reported in another study [28].

Sorafenib was approved based on a higher PFS when compared to placebo when censored at cross-over, and longer PFS when compared to placebo [12]. Sorafenib has since become the standard arm to which newer therapies are being compared [28]. The low response rate to sorafenib, however, provides the rationale for predictive biomarker studies to improve the therapeutic ratio.

Renal cell carcinomas are highly vascular. Tumor MVA is the most commonly used measure of angiogenesis, and in previous studies we showed that MVA in primary tumors is associated with decreased OS [22]. Others have confirmed this finding [29]. In a more recent publication, we found no major differences in MVA of nephrectomy specimens and matched metastatic tumors [25]. This suggests that for predictive biomarker marker studies, such as the one undertaken here, the primary tumor can be used as a surrogate for measuring vascularity in metastatic deposits. This provides a practical means to determine tumor vascularity when treating metastatic disease, as needle biopsies from metastatic sites might not yield sufficient tumor. However, seeing that sorafenib is currently used primarily in the second line setting, baseline MVA at the time of initial diagnosis might be altered. MVA might need to be reassessed at the time of initiation of a new VEGF/VEGFR inhibitor.

In our previous studies we found that MVA does not correlate well with expression levels of VEGF and its receptors. These studies were done in a large tumor cohort of over 300 cases [22]. This is likely due to the fact that additional growth factors determine vessel density, and these are likely not affected by sorafenib.

It is unclear whether sorafenib inhibits tumor growth by inhibiting angiogenesis or by direct inhibition of drug targets in tumor cells. Our results suggest that the former might be the more important mechanism of action of the drug, supported by other small studies using DCE-MRI, showing an association between baseline tumor vascularity and greater benefit from sorafenib [18]. Patients with mRCC and prior nephrectomy with available tissue can thus be assessed for likelihood of sorafenib response by a simple tissue based assay, such as the one used here, rather than by more expensive imaging modalities. We hypothesize that the inhibitory effect of sorafenib on angiogenic factors and their receptors effectively lowers MVA, resulting in decreased tumor viability, a hypothesis that is supported by Flaherty et al. who found a decrease in tumor vascularity from baseline in sorafenib-treated patients responding to therapy [18]. Highly vascular tumors might be more susceptible to sorafenib, as these tumors may be more dependent on the vasculature to proliferate.

Although it has been over nine years since sorafenib has been approved by the Food and Drug Administration, no predictive assays have been validated for this drug (or any of the other approved VEGF or VEGFR inhibitors). A clinical trial has been completed assessing the association between response to sorafenib and bevacizumab and a variety of tissue based biomarkers including MVA, as well as imaging based predictors (NCT00126503). The results have not been published. Recent studies by Zhao et al. suggests that high MVA predicts better response to bevacizumab in non small cell lung cancer [30]. VHL mutations might be associated with benefit from VEGF/VEGFR targeting drugs, and we are currently assessing the association between VHL mutations and clinical benefit from sorafenib and other drugs in RCC tumors.

In our cohort, high MVA in nephrectomy samples was associated with smaller primary tumors. A large recent study showed a distinct subpopulation of RCC patients with smaller primary tumors who developed distant metastasis [31]. This subpopulation might be the patients more likely to respond to anti-angiogenic therapy. Expression of some of the angiogenic factors studied here was associated with worse clinical features, such as poor performance status and low hemoglobin, but not with response to therapy.

Modest response rates to sorafenib have led to use of sorafenib in combinatorial studies, but a superior combination has not been identified [28]. Alternative VEGF-receptor inhibitors are available for clinical use, many with superior response rates to sorafenib. However, all are associated with toxicities, and due to the relatively favorable toxicity profile of sorafenib, the drug is still used, either in the second line setting, or for patients who do not tolerate other drugs in this class [32].

In summary, in our study we found an association between microvessel area in nephrectomy specimens and response to sorafenib. Use of MVA as a predictor of response should be validated and confirmed in additional RCC cohorts. Furthermore, our results suggest that MVA should be studied as a potential predictor of response to other anti-angiogenic therapies as well.

## Competing interests

The authors declare that they have no competing interests.

## Authors’ contributions

SAA and JAS performed the immunofluorescent staining. JAS and RLC performed the statistical analysis. LA, BE, SAA and HMK collected patient samples and compiled the clinical database. CZ and LBJ assisted in database management and compiled the biomarker database. HMK and BE oversaw the project. JAS, LBJ, RLC and HMK compiled the manuscript. All authors read and approved the final manuscript.

## Supplementary Material

Additional file 1: Table S1 Antibody source and dilution information. **Table S2.** AQUA score distributions for markers analyzed. **Table S3.** Correlations between AQUA scores and clinical variables. Click here for file
