# Supplementary material for: Microvessel area as a predictor of sorafenib response in metastatic renal cell carcinoma
Source: Cancer Cell Int. 2014 Jan 14;14:4. doi: 10.1186/1475-2867-14-4 (PMC3896780; doi:10.1186/1475-2867-14-4)
Supplement: Additional file 1: Table S1 — Antibody source and dilution information. Table S2. AQUA score distributions for markers analyzed. Table S3. Correlations between AQUA scores and clinical variables. [file 1475-2867-14-4-S1.doc]

Additional file 1

Table S1: Antibody source and dilution information

| Target Name | Source | Concentration |
| --- | --- | --- |
|  |  |  |
| B-Raf | Santa Cruz | 1-100 |
|  | (rabbit polyclonal) C-19 |  |
| C-Raf | Upstate | 1-100 |
|  | (rabbit monoclonal) 04-412 |  |
| c-KIT | Dako | 1-50 |
|  | (rabbit polyclonal) A4502 |  |
| PDGF-Rβ | BD Transduction Lab | 1-1000 |
|  | (mouse monoclonal) C-28 |  |
| VEGF-R1 | Santa Cruz | 1-200 |
|  | (rabbit polyclonal) C-17 |  |
| VEGF-R2 | Santa Cruz | 1-200 |
|  | (mouse monoclonal) A-3 |  |
| VEGF-R3 | Santa Cruz | 1-500 |
|  | (rabbit polyclonal) C-20 |  |
| VEGF | Santa Cruz | 1-500 |
|  | (rabbit polyclonal) A-20 |  |
| CD-34 | Dako | 1-50 |
|  | (mouse monoclonal) Class II Clone QBEnd 10 |  |

Table S2: AQUA score distributions for markers analyzed.

| Marker | N | AQUA Score (mean, ±SD) | Range |
| --- | --- | --- | --- |
| MVA | 97 | 6.3, ±5.5 | 0.4-29.9 |
| VEGF | 98 | 36.9, ±11.1 | 17.2-75.8 |
| VEGF-R1 | 98 | 32.7, ±8.1 | 20.8-58.5 |
| VEGF-R2 | 97 | 32.8, ±8.2 | 15.6-64.2 |
| VEGF-R3 | 95 | 50.3, ±10.7 | 28.7-89.9 |
| B-Raf | 97 | 41.7, ±13.7 | 14.7-88.8 |
| C-Raf | 96 | 34.4, ±12.8 | 10.6-79.0 |
| c-KIT | 96 | 43.7, ±13.1 | 19.5-91.2 |
| PDGF-Rβ | 96 | 34.4, ±10.8 | 11.2-78.9 |

**Table S3:** Correlations between AQUA scores and clinical variables

|  | Performance Status | | LDH | | Calcium | | Hemoglobin | |
| --- | --- | --- | --- | --- | --- | --- | --- | --- |
|  | 0 vs 1,2 | | Normal vs Elevated | | Normal vs Elevated | | Normal vs Low | |
| Marker | *t-statistic* | *p value* | *t-statistic* | *p value* | *t-statistic* | *p value* | *t-statistic* | *p value* |
| MVA | 1.494 | 0.139 | 0.982 | 0.357 | 0.664 | 0.54 | 0.061 | 0.952 |
| VEGF | 0.07 | 0.944 | 2.567 | **0.032** | 0.277 | 0.793 | 0.834 | 0.401 |
| VEGF-R1 | 0.76 | 0.449 | 1.364 | 0.207 | 0.014 | 0.99 | 1.187 | 0.242 |
| VEGF-R2 | 2.055 | **0.043** | 1.857 | 0.104 | 0.143 | 0.893 | 2.909 | **0.006** |
| VEGF-R3 | 1.08 | 0.283 | 1.605 | 0.138 | 0.585 | 0.588 | 2.191 | **0.036** |
| B-Raf | 1.914 | 0.059 | 1.239 | 0.247 | 0.037 | 0.972 | 1.01 | 0.32 |
| C-Raf | 0.174 | 0.863 | 1.172 | 0.283 | 0.112 | 0.915 | 0.666 | 0.511 |
| c-KIT | 1.955 | 0.054 | 0.586 | 0.567 | 0.14 | 0.895 | 1.612 | 0.116 |
| PDGF-Rβ | 2.313 | **0.023** | 0.067 | 0.948 | 0 | 0.999 | 2.104 | **0.044** |
